# Editorial: The Contribution of Postural Adjustments to Body Balance and Motor Performance: Volume II

**DOI:** 10.3389/fnhum.2022.910540

**Published:** 2022-05-24

**Authors:** Teddy Caderby, Paolo Cavallari, Martin Descarreaux, Eric Yiou

**Affiliations:** ^1^Laboratoire IRISSE, UFR des Sciences de l'Homme et l'Environnement, Université de la Réunion, Le Tampon, France; ^2^Human Physiology Section of the Department of Pathophysiology and Transplantation, Università Degli Studi di Milano, Milan, Italy; ^3^Department of Human Kinetics, Université du Québec à Trois-Rivières, Trois-Rivières, QC, Canada; ^4^EA 4532 Complexité, Innovation, Activités Motrices et Sportives, Université Paris-Saclay, Orsay, France; ^5^Laboratoire Complexité, Innovation et Activités Motrices et Sportives, Université d'Orléans, Orléans, France

**Keywords:** postural control, rehabilitation, innovative tools, gait, multisensory integration, motor control, human

This second volume of the Research Topic provides an up-to-date picture on how humans control balance and body motion during daily motor tasks, with a special focus on the relationship between postural adjustments, body balance and motor performance in healthy adults and individuals with various health conditions. It includes 18 contributions separated into four sections, each of them focusing on a specific aspect of balance and body motion control. In the first section, the focus is on multisensory integration in balance control during standing, sitting and gait initiation. The second section reports results of studies investigating the adaptability of gait and balance control under specific and controlled conditions in healthy individuals. The third section reports results of studies focusing on gait and balance disorders in specific clinical populations. Finally, in the last section, results of studies focusing on the development of innovative tools and methods to assess and improve gait, balance and cognitive functions in neurological patients are reported.

## Multisensory Integration in Balance Control

Balance control is a complex motor skill which involves the integration of many types of sensory information (sight, vestibular system and proprioception) and can be modified by sensory input alterations consequent to a pathology or aging processes.

Postural instability during stance with eyes closed was investigated by Sozzi et al. by using spectral analysis of center-of-pressure (COP) oscillations and different sensory conditions. While standing on foam, vision did not reduce low-frequency oscillations, while touch diminished the entire spectrum, except for the medium-high frequencies, as if sway reduction by touch would rely on rapid balance corrections.

Desgagnés et al. showed that surface anodal stimulation over the right mastoid in seated participants induced an ipsilateral body sway. Current intensity and duration affected amplitude and occurrence of the inhibitory short-latency and the excitatory medium-latency responses in lumbar erector spinae muscles; responses were amplified by right, but not left, head rotation. Vision did not influence the responses, suggesting its minimal contribution to vestibulomotor control during sitting. The lack of response reversal in the sagittal plane may reflect the biomechanical role of lumbar erector spinae to tune the lumbar lordosis during an induced body sway.

Relative contribution of sensory and motor components on postural control in young and old participants was investigated both by Lauzier et al. and Kimijanová et al. According to the Canadian group, feet soles vibration and standing on foam increased COP parameters, but foam was more effective than vibration in both groups. Conversely to foam, changes in COP parameters after vibration were no longer different between groups when correcting for the baseline levels; thus aging seems to differently affect “motor” and “sensory” components, the latter being relatively unaltered. The Slovak group showed instead that young people significantly modified anticipatory postural adjustments (APAs) during gait initiation when a previous vibratory perturbation was delivered to lower leg muscles. Sensitively scaled APAs according to the actual position of the body verticality, an effect which is absent in old participants. Significant age-related declines in APAs were observed regardless of altered proprioception. This seems to indicate that at the transition from standing to walking they probably require higher reliance on the visual input.

Since aging is known to increase the falling risk, the Sensory Organization Test (SOT) applied to old people appears to be a useful tool for assessing postural stability, risk of falling, and balance improvement during rehabilitation. Perucca et al. presented normative data of SOT for 80–84 and 85–89 years groups and concluded that the vestibular balance tended to be affected by aging more than vision and proprioception-based systems. However, Moon et al. stressed that SOT has limitations (high cost, lack of portability etc.) that hinder its use in clinical practice. Thus they developed an innovative system, called VR-ComBAT (for “Virtual Reality Comprehensive Balance Assessment and Training”), that provides a safe, feasible, and cost-effective virtual reality environment allowing the investigation of multisensory integration in balance control.

## Adaptability of Gait and Postural Control

Investigating gait and postural control adaptations under specific and controlled conditions in healthy individuals can be a relevant approach to identify solutions for the assessment and improvement of these functions in people with balance and mobility impairments. Bertrand-Charrette et al. pointed out that pain experimentally induced by electrical stimulation caused robust gait pattern adaptations in healthy participants and thus provided a valid model to study musculoskeletal pain-induced gait adaptations under controlled conditions.

During toe walking De Pieri et al. showed that healthy adults generate a larger support moment during the stance phase to maintain the knee stable compared to normal walking. These results suggest that the support moment can be an insightful parameter for assessing the muscle demand associated with gait in patients affected by toe walking, such as patients with cerebral palsy or idiopathic toe walking.

Tebbache and Hamaoui showed that during a sit-to-stand task, an increase in backrest inclination resulted in an increase in the activity level of the neck and trunk flexor muscles during the postural phase (prior to seat-off), but a decreased level activity of some lower limb muscles during the rising phase. These results, which reveal a change in muscle demand as a function of backrest inclination, may be particularly useful in developing solutions to improve sit-to-stand performance in people with muscle weakness.

## Gait and Balance Disorders in Specific Clinical Populations

Several musculoskeletal and neurological conditions can affect gait and postural control often leading to increased disability and significant reduction in quality of life and life expectancy. Consequently, much attention has been given to the investigation of both gait and postural control in patients with various neuromusculoskeletal conditions, either to establish baseline comparisons with healthy individuals, track natural history of diseases or assess clinical improvement following clinical interventions. As illustrated by the following studies, translational research including basic sciences, applied research and pragmatic clinical studies significantly contributes to the development of tailored rehabilitation strategies targeting gait and balance disorder.

Student et al. compared mean sway amplitude and mean velocity of both the COP and COM in patients with early-to-mid stage Parkinson's disease (PD), aged-matched healthy individuals and young healthy adults. Using a moving room paradigm, the authors showed that the net effect of a visual perturbation on mean COP velocity was significantly higher in patients with early-to-mid stage PD and aged-matched healthy individuals suggesting that such changes are mostly an effect of aging rather than a specific effect of early PD.

In a study investigating the task specificities of anticipatory muscle activations throughout various hand activities in subacute stroke survivors, Xia et al. showed several differences between stroke patients and healthy participants. Among the several between group differences observed, the authors reported changes in anticipatory muscle activations in the extensor carpi radialis muscle as well as changes in APAs.

Walking capacity is a critical health outcome in several patient populations with musculoskeletal or neurological diseases. Through a scoping review, Houle et al. summarized the best currently available scientific evidence regarding walking capacity in patients with lumbar spinal stenosis and associated neurogenic claudication. Their review showed on one side that several physical and psychological factors are associated with walking capacity in patients with lumbar spinal stenosis, and on the other side that assessment of walking capacity and associated physical and psychological factors lacks standardization therefore limiting stronger conclusions on the topic.

Spinocerebellar ataxia type 3 is a neurodegenerative disorder characterized by progressive ataxia, external progressive ophthalmoplegia, and other neurological manifestations. Liu et al. evaluated lower limb proprioception in 80 patients to assess the association between proprioception and clinical features of spinocerebellar ataxia type 3. Their results showed that lower limb proprioception was significantly impaired in patients with spinocerebellar ataxia type 3 and that increased impairment was associated with increasing age at onset and increasing disease duration.

Finally, Souza et al. compared the index finger kinematics during a free-endpoint whole-body reaching task and a cup-to-mouth task in participants with traumatic brachial plexus injury (uninjured limb) and healthy matched controls. Significant differences in movement parameters led the author to suggest that traumatic brachial plexus injuries lead to increased motor planning cost for the movement of the uninjured limb and that this cost is higher in tasks that require greater postural balance.

## Gait, Balance and Cognitive Rehabilitation in Patients With Parkinson's Disease and Stroke

The development of innovative tools and methods directed to assess and improve gait, balance and cognitive functions in PD patients and stroke are important for rehabilitation interventions.

Bhatt et al. developed a computer game-based rehabilitation treadmill platform for dual-task (DT) assessment and training in PD. Authors showed that this platform has the ability to repeatedly record reliable DT interference effects over time and track PD progression. The platform is directly applicable to other diseases than PD that affect gait and cognition.

Functional electrical stimulation (FES) and robot-aided stretching device are two other promising tools that may improve balance and gait function in neurological patients. Delafontaine et al. reported that bilateral applications of FES to the *tibialis anterior* muscles (ankle dorsi-flexor) may improve the capacity of PD to generate the APAs associated with gait initiation. Future studies are required before considering that this tool is valuable to optimize the effects of L-DOPA medication on gait initiation.

Zhai et al. stressed that stroke survivors with impaired control of the ankle due to stiff plantar-flexors often experience abnormal postural control, which affects balance and gait. These authors reported that robot-aided and manual ankle stretching training provided similar improvements in the ankle properties and balance post-stroke. However, only the robot-aided stretching training improved spasticity and stiffness of dorsiflexion significantly. Authors thus suggested that robot-aided rehabilitation may optimize current rehabilitation programs in stroke survivors.

Beside the use of innovative tools, physical activity is important for the rehabilitation and promotion of healthy aging. Zhang et al. defended the point-of-view that the practice of tai chi, a traditional Chinese martial art, can improve balance ability and reduce the risk of falls in people with mild to moderate PD.

In conclusion, this collection contributes to a better understanding of the basic mechanisms underlying the control of body balance and body motion during daily motor tasks. The collection also promotes the development of innovative tools and methods to assess and train gait and balance, with relevant applications in the fields of neurodegenerative conditions and rehabilitation.

## Author Contributions

All authors listed have made a substantial, direct, and intellectual contribution to the work and approved it for publication.

## Conflict of Interest

The authors declare that the research was conducted in the absence of any commercial or financial relationships that could be construed as a potential conflict of interest.

## Publisher's Note

All claims expressed in this article are solely those of the authors and do not necessarily represent those of their affiliated organizations, or those of the publisher, the editors and the reviewers. Any product that may be evaluated in this article, or claim that may be made by its manufacturer, is not guaranteed or endorsed by the publisher.

